# The Risk of Severe Fibromyalgia, Depression, Anxiety, and Insomnia Symptoms in Arab Women: An Implication of Self-Medication with Analgesics? A Cross-Sectional Study

**DOI:** 10.3390/medicina60071065

**Published:** 2024-06-28

**Authors:** Omar Gammoh, Mariam Al-Ameri, Ghaith Altaani, Ahmed Al-smadi, Reham Al-Zegoul, Talal Massad, Ahmad F. Klaib, Mervat Alsous, Ammena Y. Binsaleh, Sireen Abdul Rahim Shilbayeh

**Affiliations:** 1 Department of Clinical Pharmacy and Pharmacy Practice, Faculty of Pharmacy, Yarmouk University, Irbid 21163, Jordan; m.alameri@yu.edu.jo (M.A.-A.); g.altaani@yu.edu.jo (G.A.); mervat.alsous@yu.edu.jo (M.A.); 2 Faculty of Nursing, Al al-Bayt University, Mafraq 25113, Jordan; a.smadi@aabu.edu.jo (A.A.-s.); reham2009@aabu.edu.jo (R.A.-Z.); 3 Faculty of Medicine, The University of Jordan, Amman 11942, Jordan; massad.talal@gmail.com; 4 Information Systems Department, Faculty of Information Technology and Computer Science, Yarmouk University, Irbid 21163, Jordan; ahmad.klaib@yu.edu.jo; 5 Software Engineering Department, King Hussein School of Computing Sciences, Princess Sumaya University for Technology, Amman 11941, Jordan; 6 Department of Pharmacy Practice, College of Pharmacy, Princess Nourah bint Abdulrahman University, P.O. Box 84428, Riyadh 11671, Saudi Arabia; aysaleh@pnu.edu.sa (A.Y.B.); ssabdulrahim@pnu.edu.sa (S.A.R.S.)

**Keywords:** fibromyalgia, depression, anxiety, insomnia, women, risk factors

## Abstract

*Background and Objectives:* The investigation of the psychosomatic symptoms in women residing in developing countries is still emerging. To be precise, the prevalence and correlates of severe fibromyalgia, depression, anxiety, and insomnia are understudied in Arab women, as these symptoms could relate to improper self-medication. This study mainly investigated the association between self-medication with analgesics and fibromyalgia, depression, anxiety, and insomnia symptoms among a community-based cohort of females in Jordan. *Materials and Methods:* We used a web-based cross-sectional study design. Fibromyalgia, depression, anxiety, and insomnia were assessed using validated scales. The used over-the-counter (OTC) painkillers were recorded. *Results:* Data were analyzed from 741 women, and fibromyalgia was screened in 16.4%, depression in 37.4%, anxiety in 27.8%, and insomnia in 38.3%. Fibromyalgia was associated with “married” (OR = 1.5, 95% CI = 1.017–2.305), “using OTC acetaminophen” (OR = 1.75, 95% CI = 1.15–2.69), “using herbal remedies” (OR = 2.02, 95% CI = 1.33–3.07), and “using antiseizure medications” (OR = 2.43, 95% CI = 1.38–4.28). Severe depression was significantly associated with “age” (OR = 0.97, 95% CI = 0.96–0.99), “high school education” (OR = 1.90, 95% CI = 1.21–2.98), “smoking” (OR = 1.72, 95% CI = 1.15–2.56), “OTC acetaminophen” (OR = 1.40, 95% CI = 1.02–1.92), “OTC non-steroidal anti-inflammatory drugs” (OR = 1.75, 95% CI = 1.15–2.65), and “antiseizures” (OR = 2.19, 95% CI = 1.30–3.70). Severe anxiety was significantly associated with “smoking” (OR = 2.08, 95% CI = 1.40–3.12), “OTC acetaminophen” (OR = 1.48, 95% CI = 1.06–2.06), and “antiseizure medications” (OR = 2.04, 95% CI = 1.22–3.41). Severe insomnia was significantly associated with “age” (OR = 0.98, 95% CI = 0.96–0.99), “high school education” (OR = 1.58, 95% CI = 1.01–2.47), “smoking” (OR = 1.51, 95% CI = 1.01–2.25), “OTC non-steroidal anti-inflammatory drugs” (OR = 1.74, 95% CI = 1.13–2.64), “antiseizure medications” (OR = 1.84, 95% CI = 1.09–3.11), and “No analgesics” (OR = 0.48, 95% CI = 0.32–0.71). *Conclusions:* Self-medication with analgesics is associated with a high burden of psychosomatic symptoms in Arab women, and awareness campaigns are required to guide self-medication behavior.

## 1. Introduction

Fibromyalgia, a poly-symptomatic central pain syndrome affecting women more than men, is characterized by ongoing generalized pain, fatigue, sleep disturbances, low mood, cognitive decline, and consequently impaired daily functioning [1]. According to epidemiological studies, the worldwide prevalence of fibromyalgia is estimated to be between 2 and 6% [2,3]. The confirmatory diagnosis of fibromyalgia is complicated as it is difficult to distinguish from other conditions that have overlapping symptoms [2]. Several risk factors have been identified for fibromyalgia, including a family history of pain, being female, having a genetic susceptibility, and having other painful disorders concurrently, with incidence rates ranging from 30 to 50% at the time of diagnosis. People with fibromyalgia experience a vicious cycle of pain and poor mental health, including anxiety and depression [4]. Depression is featured by low mood and a lack of interest in joyful activities and it contributes to impaired daily functioning and could lead to suicide. The global self-reported depression prevalence is up to 34%, as reported from 2001 to 2020, with a women/men ratio of 2:1 [5,6]. In women, depression is closely related to several risk factors that include pregnancy, hormonal changes, stressful life events, family history, and chronic pain [7,8]. Anxiety disorders are a common global problem. Across all ages, the incidence in women is 2933 per 100,000 people compared with 4862 per 100,000 people in men. Therefore, women are more vulnerable to anxiety than men [9]. Notably, compared to the general population, women reporting chronic pain such as fibromyalgia patients experience mood and anxiety issues more than three times as frequently [10]. Insomnia can be defined as struggling to fall asleep, staying asleep, or waking up too early [11]. Insomnia has a wide prevalence range, as reported in the literature; it ranges from 8% to 42% [12,13]. According to the literature, insomnia is tightly associated with the physical and health status of the subjects; for example, insomnia is correlated with women reporting chronic pain symptoms and depression [14,15]. Jordan, a developing Arab Mediterranean country with a total area of 88,780 km^2^, is located to the east of the Jordan River and is surrounded by several conflict-affected countries and has received a high number of refugees since 1948 [16]. Although numerous medical and health-related publications are coming out from Jordan, the focus on women’s mental health research is still emerging; for example, some reports have highlighted mental health in pregnant women [17], and others have confirmed that women in Jordan experience more severe depression and anxiety compared to men [18]. In general, women are more likely to report pain and psychological symptoms and are more likely to consume analgesics [19,20]. However, no previous research tried to include fibromyalgia, depression, anxiety, and insomnia in one study among a cohort of healthy Jordanian women concerning self-medication with analgesics. An examination of the prevalence and the identification of the risk factors of fibromyalgia, depression, anxiety, and insomnia in this understudied population representing Arab countries is crucial to properly address these disorders by setting and implementing effective strategies that improve the daily functioning of women and their well-being in these countries. Therefore, the present study aimed to screen for the self-reported prevalence of fibromyalgia, depression, anxiety, and insomnia among Arab females of Jordan in association with self-medication with analgesics.

## 2. Materials and Methods

### 2.1. Study Design and Participants Recruitment

This is a cross-sectional study that recruited participants via online platforms based on predetermined inclusion criteria. All participants could exit the study at any time. The study was approved by the Yarmouk University IRB (committee number 175/2023). The data collection took place in August 2023. The sample size calculation based on our previous study revealed the need for the inclusion of 384 participants [21].

### 2.2. Inclusion Criteria

Only data from females with no chronic diseases, not using chronic medications, who filled the study instrument, and aged above 18 years old were included in the study.

### 2.3. Study Instrument

#### 2.3.1. Covariates

A well-structured self-administered online questionnaire was developed to address the study objectives. The demographics and clinical details consisted of questions to identify the participants’ age, current marital status, current employment status, educational level, smoking status, and the presence of chronic diseases. In addition, the participants were asked about their over-the-counter (OTC) analgesics of choice used to alleviate their muscular or fibromyalgia-like symptoms. The choices included acetaminophen (“APAP”), non-steroidal anti-inflammatory drugs (“NSAIDs”), homeopathic herbal remedies (anise, fennel, etc.), and prescribed centrally acting medication (“Rx CAM”) for any acute condition.

#### 2.3.2. Outcome Measures

##### Fibromyalgia

The screening for severity was carried out through a patient self-report survey (PSRS). This validated scale (Cronbach alpha = 0.94) [22] captures the different pain locations, covering nineteen possible tender points and the severity of symptoms based on the American College of Rheumatology 2011 guideline modification. A score reaching and exceeding 13 is considered positive for severe fibromyalgia as a self-reported symptom as in [23].

##### Depression

The translated and validated version (Cronbach alpha = 0.86) of the Patient Health Questionnaire-9 (PHQ-9) [24] was employed to screen for depression severity. This scale that captures the spectrum of depressive symptoms for the last 14 days generates a scale maximum score of 27, with a threshold score of 15 or above for severe depression [24,25,26,27].

##### Anxiety

The validated Arabic version (Cronbach alpha = 0.76) of the General Anxiety Disorder-7 (GAD-7) was used to evaluate the severity of anxiety in the participating women. This self-completed scale comprises seven elements that encompass anxiety symptoms during the past two-week period and has a cut-off score of fifteen or above to indicate severe anxiety as in [26,28,29].

##### Insomnia

Insomnia screening was pursued using the translated and validated Arabic scale of the Insomnia Severity Index (ISI-A) (Cronbach alpha = 0.82) [30]. The scale comprises seven items rated against Likert-type answers to evaluate insomnia severity [31] and has a threshold score of 15 or above for severe insomnia as in [32].

### 2.4. Statistical Analysis

Frequencies and percentages were employed to present the characteristics of the study participants. To investigate the association between the covariates and the outcome variables (fibromyalgia, depression, anxiety, and insomnia), a preliminary univariate analysis was performed for each of the four dependent variables, and all candidate variables showing a value of *p* < 0.10 were included and fed into the multivariable regression models for four dependent variables. Statistical significance was set at 2-sided *p* < 0.05 and estimates were set at 95% CIs.

## 3. Results

### 3.1. Response Rate

A total of 1100 females were approached; 241 were excluded due to their diagnosis with chronic diseases, and 118 were excluded because of their incomplete data. Therefore, data were analyzed from 741 participants.

### 3.2. Study Sample Characteristics

The majority of our sample, 663 (89.5%), were females aged below 50 years, 443 (59.5%) were single, 541 (72.6%) were unemployed, and 617 (82.8%) were non-smokers. In regards to the over-the-counter analgesics used to alleviate fibromyalgia-like symptoms, 397 (53.3%) reported using APAP, 206 (27.7%) reported using homeopathic herbal remedies, 118 (15.8%) reported using NSAIDs, 69 (6.69%) reported using “Rx for CAM”, and 194 (26.0%) reported not using analgesics, as in Table 1.

### 3.3. Prevalence of Fibromyalgia, Depression, Anxiety and Insomnia

The prevalence of fibromyalgia was estimated via a patient self-report survey; a total of 122 participants (16.4%) reported having scores above the threshold for fibromyalgia symptoms. Depression was assessed using the PHQ-9 scale; 279 participants (37.4%) reported scores corresponding to severe depression. Anxiety was evaluated using the GAD-7 scale, with 207 participants (27.8%) reporting scores corresponding to severe anxiety, and insomnia was evaluated using the ISI-A scale, with 285 participants (38.3%) reporting scores corresponding to severe insomnia. Refer to Table 2.

### 3.4. Determinants of Fibromyalgia, Depression, Anxiety, and Insomnia

An assessment of the possible determinants for fibromyalgia, depression, anxiety, and insomnia was determined using an initial univariate binary logistic regression model to determine the potential confounders, and afterward, a multivariate binary logistic regression model was built for each of the outcome variables.

The multivariable regression model for fibromyalgia was finally adjusted for “marital status”, “using OTC APAP”, “using homeopathy herbal remedies”, and “using Rx CAM” and revealed that fibromyalgia symptoms were significantly associated with married (OR = 1.5, 95% CI = 1.017–2.305, *p* = 0.04), “using OTC APAP” (OR = 1.75, 95% CI = 1.15–2.69, *p* = 0.01), “using homeopathy herbal remedies” (OR = 2.02, 95% CI = 1.33–3.07, *p* = 0.001), and “using Rx CAM” (OR = 2.43, 95% CI = 1.38–4.28, *p* = 0.002).

The multivariable regression model for depression was finally adjusted for “age”, “education level”, “smoking status”, “OTC APAP”, “OTC NSAIDs”, and “Rx CAM” and revealed that severe depression was significantly associated with “age” (OR = 0.97, 95% CI = 0.96–0.99, *p* < 0.001), “high school education” (OR = 1.90, 95% CI = 1.21–2.98, *p* = 0.005), “smoking” (OR = 1.72, 95% CI = 1.15–2.56, *p* = 0.008), “OTC APAP” (OR = 1.40, 95% CI = 1.02–1.92, *p* = 0.03), “OTC NSAIDs” (OR = 1.75, 95% CI = 1.15–2.65, *p* = 0.009), and “Rx CAM” (OR = 2.19, 95% CI = 1.30–3.70, *p* = 0.003).

The multivariable regression model for anxiety was finally adjusted for “smoking status”, “OTC APAP”, and “Rx CAM” and revealed that severe anxiety was significantly associated with “smoking” (OR = 2.08, 95% CI = 1.40–3.12, *p* < 0.001), “OTC APAP” (OR = 1.48, 95% CI = 1.06–2.06, *p* = 0.02), and “Rx CAM” (OR = 2.04, 95% CI = 1.22–3.41, *p* = 0.007).

The multivariable regression model for insomnia was finally adjusted for “age”, “education level”, “smoking status”, “OTC NSAIDs”, “Rx CAM”, and “No analgesics” and revealed that severe insomnia was significantly associated with “age” (OR = 0.98, 95% CI = 0.96–0.99, *p* < 0.01), “high school education” (OR = 1.58, 95% CI = 1.01–2.47, *p* = 0.04), “smoking” (OR = 1.51, 95% CI = 1.01–2.25, *p* = 0.04), “OTC NSAIDs” (OR = 1.74, 95% CI = 1.13–2.64, *p* = 0.01), “Rx CAM” (OR = 1.84, 95% CI = 1.09–3.11, *p* = 0.02), and “No analgesics” (OR = 0.48, 95% CI = 0.32–0.71, *p* < 0.01). See Table 3 below.

## 4. Discussion

The present study aimed to screen for the self-reported prevalence of fibromyalgia, depression, anxiety, and insomnia among females in Jordan and also to examine whether self-medication with analgesics was related to poor outcomes for these symptoms. We report high rates of these symptoms that were associated primarily with smoking and the improper use of OTC analgesics.

Our findings reported the prevalence of fibromyalgia to be 16%; this is considered higher compared to previous studies where fibromyalgia prevalence ranged between 6% and 15% under some circumstances [3]. This variation in the results can be mainly attributed to the scale used, the sample type employed in the study, and the extent of the accuracy of the data. The exact diagnosis of fibromyalgia is challenging and requires specialists, laboratory tests, and other resources, while cohort studies rely on self-administered scales to capture an estimation of the prevalence.

In our study, women with positive fibromyalgia screening were married. Marital status has been studied as a risk factor for fibromyalgia; according to the results, married women reported higher odds for fibromyalgia [33]. Moreover, another study suggested that adjustments in marital status among women with fibromyalgia increased the likelihood of severe depression [34]. Married women are always under higher stress and physical demands due to household responsibilities compared to single women.

The findings of our study revealed that OTC NSAIDs and homeopathic herbal remedies were associated with positive fibromyalgia screening. This finding underscores the deviation from the ACR guidelines due to the lack of community awareness about fibromyalgia. Fibromyalgia, as a central pain, is related to neuropathic origin; therefore, the optimal pharmacological treatment should comprise medications such as antiseizure medications and/or specific classes of antidepressants. Also, the use of “Rx CAM” in about 6% of the sample was associated with fibromyalgia. This can be explained by the fact that some participants are currently using CAM for fibromyalgia based on a proper diagnosis, for acute low back pain, or for other acute indications [35].

Our findings demonstrated that 37% of our sample reported severe depression. According to one cross-sectional pilot study conducted ten years ago, depression prevailed in 51% of working women [36]. Similar studies coming from Jordan focused on perinatal depression in Jordanian women. However, our study is the latest to examine depression among healthy women regardless of hormonal status. Of importance, smoker women who only received a high school education were at higher odds for severe depression. Our findings are consistent with previous studies; for example, depression is suggested to prevail twice as much in current smokers compared to non-smokers [37]. Conversely, depression is a leading factor in smoking initiation [38].

In addition, we report an association between using APAP and NSAIDs with severe depression. As mentioned above, the lack of proper pain management contributes to depression. It has been suggested that subjects with depression have altered pain sensitivity, and it has been confirmed that chronic pain mediates new depression episodes, and depression significantly predicts the onset of new chronic pain [8].

Several studies have investigated factors associated with anxiety, and the results of our study are consistent with many studies reporting a positive association between smoking and anxiety [39,40,41]. Interestingly, anxiety was positively associated with participants taking acetaminophen, a commonly used over-the-counter pain reliever, which describes that people with symptoms of anxiety have pain. The association between anxiety and the use of centrally acting drugs may be related to the side effects of drugs such as antiepileptic drugs and centrally acting muscle relaxants. Another explanation is that due to severe reported pain, these women suffer from more anxiety [42,43].

In this study, insomnia was assessed using the Insomnia Severity Index (ISI). According to this index, 38.3% reported severe clinical insomnia, a prevalence that approached the reported prevalence in previous studies [13]. It is established that insomnia prevalence varies depending on the measurement tools used, the study type, and the sample size [12].

The results of this study revealed that older age, low educational level, being a smoker, chronic OTC NSAID use, and a need for CAM therapy were risk factors for insomnia. Older age as a risk factor for insomnia is supported by several studies in the literature [44,45,46,47]. Low education level and smoking have been demonstrated to be risk factors for insomnia, and this is supported by several studies. We suggest that women with low education do not seek professional medical care. Also, smoking exacerbates insomnia at least through the induction of biochemical alternations that affect the circadian cycle [48,49,50]. Moreover, analgesic use such as NSAIDs indicates the presence of pain, which is a significant risk factor for insomnia according to several sources [14,44,45].

To the best of our knowledge, this is the first study that explores the rates of fibromyalgia, depression, anxiety, and insomnia in a cohort of Arab women from Jordan. This novel investigation underscores the importance of focusing on the overlooked symptoms of women, and it also exhibits several strengths such as the large sample size, the validated scales, the sample type, and the robust statistical analysis. On the other hand, the outcome variables were measured through self-administered scales that could lead to some exaggerated or biased results. Therefore, future works should recruit specialized psychiatrists to provide profound and comprehensive mental and physical health assessments for participants.

## 5. Conclusions

The Arab women of Jordan experience a high prevalence of self-reported fibromyalgia, depression, anxiety, and insomnia symptoms that are associated with improper self-medication with analgesics. Community-based awareness campaigns are required to properly educate women about the proper self-care procedures regarding the use of self-medications and about the psychosomatic spectrum of symptoms that affect their well-being.

## Figures and Tables

**Table 1:** Demographic and background variables within the study sample, n = (741).

Distribution of the Participants’ Characteristics n = (741)	
Variable	n (%)
Age	
Less than 50 years	663 (89.5)
50 years and above	78 (10.5)
Marital status	
Single	443 (59.5)
Married	302 (40.5)
Education	
High school	102 (13.7)
University	643 (86.3)
Occupation	
Unemployed	541 (72.6)
Employed	204 (27.4)
Smoking status	
Non-smoker	617 (82.8)
Smoker	128 (17.2)
Analgesics used	
OTC APAP	397 (53.3)
OTC NSAIDs	118 (15.8)
Homeopathic herbal remedies	206 (27.7)
Rx for CAM	69 (6.69)
No analgesics	194 (26.0)

OTC: over the counter, APAP: acetaminophen, NSAIDs: non-steroidal anti-inflammatory drugs, CAM: centrally acting medications.

**Table 2:** The prevalence of fibromyalgia, depression, anxiety, and insomnia among the study sample using self-reported surveys.

The Prevalence of Severe Fibromyalgia, Depression, Anxiety, and Insomnia Symptoms (n = 741)	
Outcome Variable	n (%)
Fibromyalgia assessed using PSRS	
Below threshold	623 (83.6)
Above threshold	122 (16.4)
Severe depression assessed using PHQ-9	
Below threshold	466 (62.6)
Above threshold	279 (37.4)
Severe anxiety assessed using GAD-7	
Below threshold	538 (72.2)
Above threshold	207 (27.8)
Severe insomnia assessed using ISI-A	
Below threshold	460 (61.7)
Above threshold	285 (38.3)

Fibromyalgia was screened via PSRS using a cut-off score of >13, severe depression was assessed using PHQ-9 with a cut-off score of >14, severe anxiety was screened using GAD-7 with a cut-off score of >14, and severe insomnia was assessed using the ISI-A scale, using a cut-off score of >14.

**Table 3:** Univariate and multivariate regression models for the four dependent variables.

The Association between the Independent Variables and Each of the Four Dependent Variables						
Fibromyalgia						
	Univariate Analysis			Multivariate Analysis		
Factor	OR	95% CI	*p*	aOR	95% CI	*p*
Age	1.01	0.99–1.02	0.32			
Marital status (married) *	1.72	1.16–2.53	0.007	1.53	1.02–2.31	0.04
Education (university)	0.72	0.43–1.22	0.22			
Occupation (employed)	1.08	0.70–1.66	0.72			
Smoking (smoker)	0.93	0.55–1.57	0.80			
OTC APAP *	2.00	1.33–3.01	0.001	1.76	1.15–2.69	0.01
OTC NSAIDs	1.87	1.16–3.00	0.009			
Herbal remedies *	2.26	1.51–3.38	<0.001	2.02	1.33–3.06	0.001
Rx for CAM *	2.91	1.69–5.02	<0.001	2.43	1.38–4.28	0.002
No analgesics	0.32	0.19–0.61	<0.001			
**Severe depression**						
	**Univariate analysis**			**Multivariate analysis**		
Factor	OR	95% CI	*p*	aOR	95% CI	*p*
Age *	0.98	0.97–0.99	0.006	0.97	0.96–0.98	<0.001
Marital status (married)	0.73	0.53 -0.99	0.044			
Education (high school) *	1.66	1.09–2.52	0.018	1.90	1.21–2.97	0.005
Occupation (employed)	0.78	0.55–1.09	0.15			
Smoking (smoker) *	2.01	1.37–2.96	<0.001	1.72	1.15–2.55	0.008
OTC APAP *	1.29	0.96–1.75	0.08	1.40	1.02–1.91	0.036
OTC NSAIDs *	1.85	1.24–2.75	0.002	1.74	1.15–2.64	0.009
Herbal remedies	1.28	0.92–1.78	0.13			
Rx for CAM *	2.07	1.25–3.41	0.004	2.19	1.29–3.71	0.003
No analgesics	0.67	0.47–0.96	0.030			
**Severe anxiety**						
	**Univariate analysis**			**Multivariate analysis**		
Factor	OR	95% CI	*p*	aOR	95% CI	*p*
Age	0.99	0.98–1.00	0.33			
Marital status (married)	0.92	0.66–1.28	0.62			
Education (university)	0.66	0.42–1.03	0.07			
Occupation (employed)	0.88	0.61–1.27	0.50			
Smoking (smoker) *	2.12	1.43–3.15	<0.001	2.09	1.39–3.121	<0.001
OTC APAP *	1.45	1.04–2.00	0.02	1.48	1.06–2.06	0.020
OTC NSAIDs	1.61	1.06–2.45	0.02			
Herbal remedies	1.24	0.87–1.77	0.21			
Rx for CAM *	2.16	1.30–3.59	0.003	2.04	1.21–3.41	0.007
No analgesics	0.55	0.37–0.82	0.003			
**Severe insomnia**						
	**Univariate analysis**			**Multivariate analysis**		
Factor	OR	95% CI	*p*	aOR	95% CI	*p*
Age *	0.99	0.98–1.00	0.18	0.98	0.97–0.99	0.016
Marital status (married)	1.03	0.76–1.39	0.82			
Education (high school) *	1.45	0.95–2.21	0.08	1.58	1.01–2.47	0.045
Occupation (employed)	0.70	0.50–0.98	0.04			
Smoking (smoker) *	1.79	1.22–2.63	0.003	1.51	1.01–2.25	0.045
OTC APAP	1.62	1.20–2.19	0.001			
OTC NSAIDs *	2.17	1.46–3.24	<0.001	1.73	1.13–2.64	0.010
Herbal remedies	1.61	1.16–2.23	0.004			
Rx for CAM *	2.12	1.29–3.50	0.003	1.85	1.09–3.10	0.021
No analgesics *	0.42	0.29–0.60	<0.001	0.48	0.32–0.70	<0.001

OR: Odds ratio, aOR: adjusted odds ratio, CI: confidence interval, * *p* < 0.05.

## Data Availability

Data will be available from the corresponding author upon request.
